# Altered Developmental Expression of the Astrocyte-Secreted Factors Hevin and SPARC in the Fragile X Mouse Model

**DOI:** 10.3389/fnmol.2017.00268

**Published:** 2017-08-29

**Authors:** Jessica Wallingford, Angela L. Scott, Kelly Rodrigues, Laurie C. Doering

**Affiliations:** ^1^McMaster Integrative Neuroscience Discovery and Study (MiNDS), McMaster University Hamilton, ON, Canada; ^2^Department of Pathology and Molecular Medicine, McMaster University Hamilton, ON, Canada

**Keywords:** astrocyte, development, Fragile X syndrome, hevin, SPARC, synapse

## Abstract

Astrocyte dysfunction has been indicated in many neurodevelopmental disorders, including Fragile X Syndrome (FXS). FXS is caused by a deficiency in fragile X mental retardation protein (FMRP). FMRP regulates the translation of numerous mRNAs and its loss disturbs the composition of proteins important for dendritic spine and synapse development. Here, we investigated whether the astrocyte-derived factors hevin and SPARC, known to regulate excitatory synapse development, have altered expression in FXS. Specifically, we analyzed the expression of these factors in wild-type (WT) mice and in *fragile X mental retardation 1 (Fmr1)* knock-out (KO) mice that lack FMRP expression. Samples were collected from the developing cortex and hippocampus (regions of dendritic spine abnormalities in FXS) of *Fmr1* KO and WT pups. Hevin and SPARC showed altered expression patterns in *Fmr1* KO mice compared to WT, in a brain-region specific manner. In cortical tissue, we found a transient increase in the level of hevin in postnatal day (P)14* Fmr1* KO mice, compared to WT. Additionally, there were modest decreases in *Fmr1* KO cortical levels of SPARC at P7 and P14. In the hippocampus, hevin expression was much lower in P7 *Fmr1* KO mice than in WT. At P14, hippocampal hevin levels were similar between genotypes, and by P21 *Fmr1* KO hevin expression surpassed WT levels. These findings imply aberrant astrocyte signaling in FXS and suggest that the altered expression of hevin and SPARC contributes to abnormal synaptic development in FXS.

## Introduction

Fragile X syndrome (FXS), the most common inherited, single-gene cause of autism spectrum disorders (ASD) and cognitive impairment (reviewed in Lubs et al., 2012; Wang et al., 2012), is a neurodevelopmental disorder characterized by a deficiency in the fragile X mental retardation protein (FMRP; reviewed in Bhakar et al., 2012). Individuals with FXS can exhibit mild to severe cognitive impairment, autistic behaviors, attention deficits, susceptibility to seizures, hypersensitivity to sensory stimuli, disrupted sleep, as well as an assortment neurobiological abnormalities (Comery et al., 1997; Nimchinsky et al., 2001; Beckel-Mitchener and Greenough, 2004; Kronk et al., 2010; Marco et al., 2011). Numerous studies examining the altered neurobiology in FXS have focused on the changes at the level of dendritic spines, the primary site for excitatory connections between neurons (Ivanov et al., 2009). The absence of FMRP in FXS has been associated with altered synapse structure, number and function (reviewed in Pfeiffer and Huber, 2009). Studies of FXS in humans or animal models have described a significant increase in the number of dendritic spines associated with FXS, with a greater proportion of immature spine phenotypes (i.e., long, thin, tortuous dendritic spines; Comery et al., 1997; Irwin et al., 2001; Nimchinsky et al., 2001). Under normal conditions, FMRP is expressed in neurons (Sidorov et al., 2013), oligodendrocyte precursor cells (Wang et al., 2004), and astrocyte cell lineages (Pacey and Doering, 2007) where it influences synaptic development through its ability to bind, transport, and regulate the local translation of several mRNAs corresponding to synaptic proteins (reviewed in Bhakar et al., 2012).

Recently, astrocytes have emerged in the literature as important regulators of synapse development and have been shown to promote both synapse formation and maturation (reviewed in Allen, 2013; Chung et al., 2015). For example, astrocyte-secreted factors, such as hevin (also known as synaptic cleft-1 or SPARC-like 1) and SPARC, have been implicated in governing the formation of excitatory synapses within the brain (Kucukdereli et al., 2011; Risher et al., 2014; Singh et al., 2016). In cultured retinal ganglion cells (RGCs) with enhanced expression of the matricellular protein hevin, a known target of FMRP (Darnell et al., 2011), there was a significant increase in synapse number (Kucukdereli et al., 2011). Likewise, the prevention of hevin expression in knock-out (KO) mice models causes a decrease in RGC-collicular synapses *in vivo*. Alternatively, Kucukdereli et al. (2011) demonstrated that in contrast to hevin, SPARC negatively regulates the formation of excitatory synapses by inhibiting the synaptogenic function of hevin, revealing an antagonistic relationship between these two factors. More recently, hevin has been shown to function as a trans-synaptic linker between presynaptic neurexin-1α and post-synaptic-1B (Singh et al., 2016). In this way, hevin assists in the formation of synapses expressing this particular neurexin and neuroligin pair, a category that includes both thalamocortical synapses and RGC-collicular synapses.

Given the respective roles known for hevin and SPARC in synapse development, aberrant expression of these astrocyte-secreted factors could account for the abnormal development and maturation of excitatory synapses in FXS. Here, we compared the developmental (postnatal day [P]7–P21) expression of hevin and SPARC in wild-type (WT) mice and mice that do not express FMRP (*Fragile X mental retardation 1* [*Fmr1*] KO; Bakker et al., 1994) across two brain regions with high levels of FMRP (cortex and hippocampus; Bakker et al., 2000). Additionally, we examined WT levels of FMRP during the same postnatal period (P7–P21) as well as thalamocortical synapse number in co-cultures containing either WT neurons and WT astrocytes or WT neurons and KO astrocytes. Importantly, our findings demonstrated that the expression of hevin and SPARC is dysregulated in both cortical and hippocampal regions with FXS. Thus, it is likely that astrocyte-mediated mechanisms significantly contribute to the neurobiological deficits associated with FXS.

## Materials and Methods

### Animals

WT and *Fmr1* KO mice (FVB.129P2[B6]-*Fmr1*^tm1Cgr^) were housed and bred in the McMaster University Central Animal Facility. All experiments and animal-handling procedures followed the guidelines set by the Canadian Council on Animal Care and were approved by the McMaster Animal Research Ethics Board (AUP 13-12-49).

### Genotyping

The tails from eight randomly selected pups from a pool of pups at ages P7, P14 or P21 (4 pups from each genotype, WT and *Fmr1* KO) were collected and the genotypes of the mice were confirmed for each group via PCR (data not shown). Segments of tails 0.5–1 cm in length were each combined with 100 μl of Extraction Solution (catalog#: E7526; Sigma-Aldrich) and 25 μl of Tissue Preparation Solution (catalog#: T3073; Sigma-Aldrich). Samples were incubated for 10 min at 55°C and then for 3 min at 95°C. Following these incubations, 100 μl of Neutralization Solution B (catalog#: N3910; Sigma-Aldrich) was added to each sample. To perform PCR, REDExtract-N-Amp PCR Reaction Mix (catalog#: R4775; Sigma-Aldrich) was added to each sample along with the following primers (with final primer concentrations of approximately 1 μM): CAC GAG ACT AGT GAG ACG TG (mutant forward; primer oIMR2060; Jackson Laboratory, Bar Harbor, ME, USA), TGT GAT AGA ATA TGC AGC ATG TGA (WT forward; primer oIMR6734; Jackson Laboratory), CTT CTG GCA CCT CCA GCT T (common; primer oIMR6735; Jackson Laboratory). Following PCR, the amplified DNA samples were run through a 2% agarose gel. Gels were imaged using SYBR Safe DNA Gel Stain (Invitrogen) and a ChemiDoc Imaging System (Bio-Rad).

### Cortical and Hippocampal Tissue Isolation for Western Blotting

WT and* Fmr1* KO male pups were decapitated at the age of P7, P14 and P21 and whole brains were extracted. Extracted brains were immediately placed into ice-cold, sterile, 0.01 M PBS and cortical and hippocampal tissue was dissected from each brain. Samples were immediately placed into separate microcentrifuge tubes, snap-frozen on dry ice, and stored at −80°C. Each sample of cortical or hippocampal tissue consisted of tissue from a single hemisphere.

Samples intended for hevin or FMRP analysis were mechanically homogenized on ice in lysis buffer (0.05 M Tris [pH 7.5], 0.5% Tween-20, 10 mM EDTA, Roche ULTRA protease inhibitor tablet, Roche PhosSTOP phosphatase inhibitor tablet). Homogenates were left on ice for 15 min and then centrifuged at 2350× *g* for 10 min at 4°C. Samples intended for SPARC analysis were mechanically homogenized on ice in RIPA buffer (150 mM NaCl, 1% NP40, 0.5% Deoxycholic Acid, 0.1% SDS, 50 mM Tris [pH 8.0], Roche ULTRA protease inhibitor tablet, Roche PhosSTOP phosphatase inhibitor tablet). Homogenates were left on ice for 1 h and then centrifuged at 16,000× *g* for 15 min at 4°C. The protein concentration of each supernatant was determined by a DC protein assay (Bio-Rad, Mississauga, ON, Canada). Samples were aliquoted and stored at −80°C.

### Cortical Astrocyte Isolation via Magnetic-Activated Cell Sorting (MACS) for Western Blotting

WT and *Fmr1* KO pups at age P14 were decapitated, whole brains were extracted, placed in ice-cold, calcium and magnesium-free Hanks buffered saline solution (CMF-HBSS), and cortical tissue was isolated from each brain. Each collected sample consisted of tissue from 2.5 cortices. Tissue and CMF-HBSS were transferred to collection tubes containing 8 mL CMF-HBSS and subsequently treated with 1.5 mL DNase (Gold Biotechnology, St. Louis, MO, USA) and 1.5 mL 2.5% trypsin (Life Technologies, Carlsbad, CA, USA). Cell suspensions were then incubated for 5 min at 37°C, after which they were triturated using a 10 mL serological pipette (Falcon, Durham, NC, USA). Cell suspensions were incubated again for 5 min at 37°C and then triturated using a 5 mL serological pipette (Falcon). The cell suspensions were then passed through a 70 μL cell strainer and centrifuged at 150× *g* for 5 min. Cells were re-suspended in 1800 mL of PBS (pH 7.4) containing 0.5% BSA.

In order to remove myelin debris from each sample, cell suspensions were first magnetically labeled via 15-min incubation at 4°C with 200 μL of Myelin Removal Beads II (catalog#: 130-096-731; Miltenyi Biotec, Bergisch Gladbach, Germany). Cells were then washed with 18 mL of PBS with 0.5% BSA and centrifuged at 150× *g* for 10 min. Cells were then re-suspended in 2000 μL of PBS with 0.5% BSA and passed through a MACS MS column (Miltenyi Biotec) that was mounted within the magnetic field of a MACS separator (Miltenyi Biotec). The negative fraction from each cell suspension, containing unlabeled cells, was collected for the subsequent isolation of astrocytes using an Anti-Astrocyte Cell Surface Antigen-2 (ACSA-2) Microbead Kit (catalog#: 130-097-678; Miltenyi Biotec). Of note, a maximum of 1 × 10^7^ cells/sample were used for the next steps of the astrocyte isolation protocol.

Cell suspensions lacking myelin debris were next centrifuged at 150× *g* for 10 min and re-suspended in 80 μL of PBS with 0.5% BSA with an additional 10 μL of Fc receptor Blocking Reagent (catalog#: 130-097-678; Miltenyi Biotec). Cell suspensions were incubated at 4°C for 10 min. Following this incubation, 10 μL of Anti-ASCA-2 Microbeads (catalog#: 130-097-678; Miltenyi Biotec) were added to each sample and incubated again at 4°C for 15 min. Cells were then washed with 2 mL of PBS with 0.5% BSA and centrifuged at 150× *g* for 10 min. The pellet was re-suspended in 500 μL of PBS with 0.5% BSA and the cell suspension was then passed through a MACS MS column mounted within the magnetic field of a MACS separator. The positive fraction from each sample, containing magnetically-labeled cells, was collected and centrifuged at 150× *g* for 10 min. The supernatant was removed and the cells were immediately flash frozen using isopentane and stored at −80°C. Cells were later homogenized in lysis buffer (0.05 M Tris [pH 7.5], 0.5% Tween-20, 10 mM EDTA, Roche ULTRA protease inhibitor tablet, Roche PhosSTOP phosphatase inhibitor tablet) and the protein concentration of each sample was determined by a DC protein assay (Bio-Rad). The homogenized samples were then aliquoted and stored at −80°C.

### Primary Cortical Astrocyte Cultures

Isolation and establishment of cortical astrocytes was carried out according to a protocol previously described by our laboratory (Jacobs and Doering, 2009). Cortical astrocytes were isolated from four WT or *Fmr1* KO pups at P1 or P2 and grown in T75 tissue culture flasks in minimum essential media (Invitrogen, Carlsbad, CA, USA) supplemented with 6% glucose and 10% horse serum (Invitrogen). Cultures were maintained for approximately 1 week at 37°C and 5% CO_2_. Cells were then removed from the T75 tissue culture flasks and re-plated onto coverslips coated with Poly-L-Lysine (Sigma-Aldrich, St. Louis, MO, USA; 1 mg/mL) and laminin (Invitrogen; 0.1 mg/mL) at a density of 5000 cells per coverslip. Cells were maintained on coverslips for 2 days *in vitro* for subsequent immunocytochemical processing or for astrocyte-neuron co-culture and subsequent immunocytochemical processing.

### Cortical and Thalamic Neuron and Cortical Astrocyte Co-Cultures with MACS

WT and *Fmr1* KO cortical astrocytes were plated onto coverslips coated with Poly-L-Lysine (Sigma-Aldrich; 1 mg/ml) and laminin (Invitrogen; 0.1 mg/mL) at a density of 5000 cells per coverslip and maintained for 2 days *in vitro* in minimal essential media (Invitrogen) supplemented with 6% glucose (Sigma-Aldrich) and 10% horse serum (Invitrogen). After 2 days this media was switched to neural maintenance media (NMM) composed of minimal essential media (Invitrogen) supplemented with 6% glucose (Sigma-Aldrich), 1% N2 supplement (Invitrogen), and 1 mM sodium pyruvate (Invitrogen). The following day, cortical and thalamic tissue was isolated from 5–6 WT pups aged P1 or P2. Cortical and thalamic tissue was dissociated using a neural tissue dissociation kit (catalog#: 130-092-628; Miltenyi Biotec). Following dissociation, cortical and thalamic cells were re-suspended in 80 μl of PBS with Mg^2+^ and Ca^2+^ and 0.5% BSA. Cells suspensions were then incubated with a biotin-antibody cocktail (catalog#: 130-098-754; Miltenyi Biotec). Cell suspensions were then washed with PBS with Mg^2+^ and Ca^2+^ and 0.5% BSA and centrifuged for 200× *g* for 10 min. Cells were re-suspended in 80 μl of PBS with Mg^2+^ and Ca^2+^ and 0.5% BSA and magnetically labeled with anti-biotin microbeads (catalog#: 130-098-754; Miltenyi Biotec) that would label non-neuronal cells within the suspension. These cell suspensions were then passed twice through a MACS MS column (Miltenyi Biotec) that was mounted within a magnetic field (MACS separator, Miltenyi Biotec). The negative fraction from each suspension, containing unlabeled cells, was collected and plated at a density of 10,000 cells per well with the previously plated astrocytes (Figure 3). Each neuronal suspension from one litter was always split and plated onto one independent WT astrocyte culture and one independent *Fmr1* KO culture in order to compare growth and synaptic development in a paired manner. This process was repeated across four independent experiments. Co-cultures were maintained in NMM for 14 days at 37°C and 5% CO_2_ and then processed for immunocytochemical analysis.

**Figure 1:**
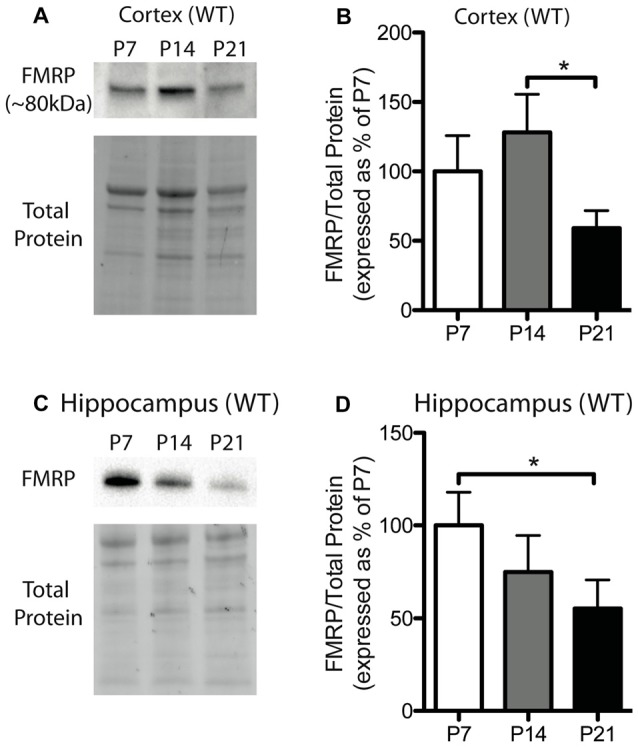
Fragile X mental retardation protein (FMRP) expression is developmentally regulated in the cortex and hippocampus. **(A)** A representative Western blot showing FMRP (~80 kDa) in wild-type (WT) cortical samples (30 μg of protein per lane) from postnatal day (P) 7, P14 and P21 mice, as well as the total protein within each lane. **(B)** FMRP expression in the cortex of WT mice at P7 (white; *n* = 8), P14 (gray; *n* = 4), and P21 (black; *n* = 8). Bands representing FMRP were normalized against the total protein within the same lane on the membrane and a cross gel control, and then expressed as a percentage of P7 FMRP. **(C)** A representative Western blot showing FMRP expression in WT hippocampal samples (30 μg of protein per lane) from P7, P14 and P21 mice, as well as the total protein within each lane. **(D)** FMRP expression in the hippocampus of WT mice at P7 (white; *n* = 6), P14 (gray; *n* = 6), and P21 (black; *n* = 6). Statistical differences were denoted with a single asterisk, *P* < 0.05.

**Figure 2:**
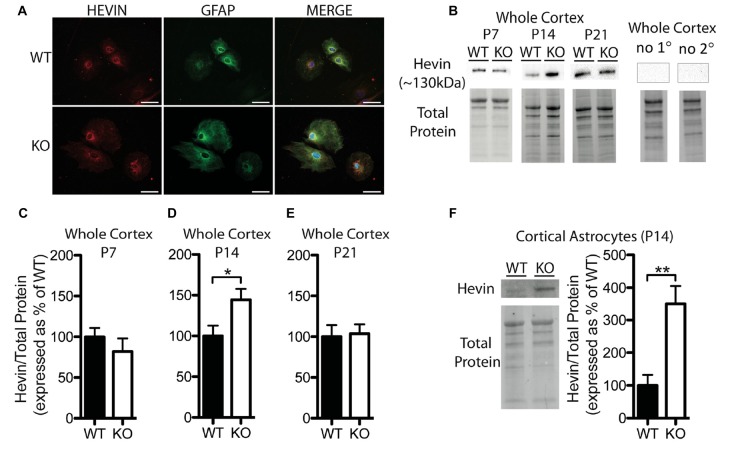
Hevin expression is altered at postnatal day (P) 14 in the cortex of *Fmr1* knock-out (KO) mice. **(A)** Cultured cortical astrocytes co-labeled with anti-glial fibrillary acidic protein (GFAP; green) and anti-hevin (red) after 2 days *in vitro*. Nuclei were stained with 4′,6-diamidino-2-phenylindole (blue). Images were obtained using a 40x objective with a Zeiss Axioimager M2. Scale bars = 50 μm. **(B)** Representative western blots showing hevin (~130 kDa) in cortical samples (30 μg of protein per lane) from P7, P14 and P21 WT and *Fmr1* KO mice, as well as the corresponding total protein within each lane. Negative controls that were run using P14 WT whole cortical tissue with either no primary antibody or no secondary antibody are shown. **(C–E)** Hevin expression in the cortex of WT (black; *n* = 8) and *Fmr1* KO (white; *n* = 8) mice at P7, P14 and P21. Bands representing hevin were normalized against the total protein within the same lane on the membrane, and were then expressed as a percent of the average level of hevin in the WT group. **(F)** Hevin expression in cortical astrocytes isolated from P14 WT (black; *n* = 4) and *Fmr1* KO (white; *n* = 4) mice. Immediately to the left of the graph is shown a representative Western blot with bands corresponding to hevin from P14 WT and *Fmr1* KO cortical astrocyte samples (10 μg of protein per lane), as well as the corresponding total protein. Statistical differences were denoted with either a single asterisk, *P* < 0.05, or a double asterisks, *P* < 0.01.

**Figure 3:**
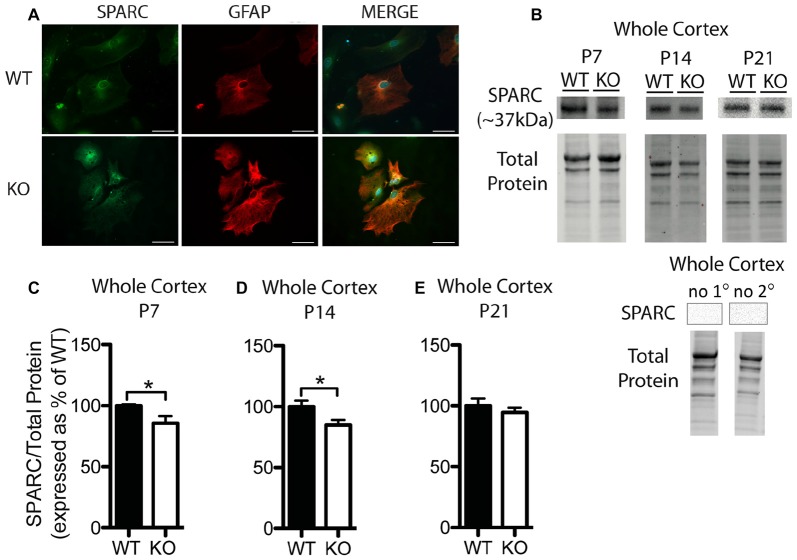
SPARC expression is altered at postnatal day (P) 7 and P14 in the cortex of *Fmr1* KO mice.** (A)** Cultured cortical astrocytes co-labeled with anti-GFAP (red) and anti-SPARC (green) after 2 days *in vitro*. Nuclei were stained with 4′,6-diamidino-2-phenylindole (blue). Images were obtained using a 40x objective with a Zeiss Axioimager M2. Scale bars = 50 μm. **(B)** A representative western blot shows bands at ~37 kDa corresponding to SPARC in cortical samples (30 μg of protein per lane) from P7, P14 and P21 WT and *Fmr1* KO mice, as well as the total protein within each lane. Negative controls that were run using P21 WT whole cortical tissue with either no primary antibody or no secondary antibody are shown.** (C–E)** SPARC expression in the cortex of WT (black, *n* = 8) and *Fmr1* KO (white, *n* = 8) mice at P7, P14 and P21, respectively. Bands representing SPARC were normalized to total protein within the same lane on the membrane and across gel controls, then expressed as a percent of the average level of SPARC in the WT group. Statistical differences were denoted with a single asterisk, *P* < 0.05.

### Immunocytochemistry

Immunocytochemistry was carried out with primary cortical astrocyte cultures following a protocol previously described by Cheng et al. (2016). The following antibodies were used: rabbit anti-glial fibrillary acidic protein (GFAP; 1:500; catalog#: Z0334; Dako, Burlington, ON, Canada), chicken anti-GFAP (1:2000; catalog#: CH22102; Neuromics, Minneapolis, MN, USA) rabbit anti-hevin antibody (1:100; catalog#: bs-6110R; Bioss, Woburn, MA, USA), goat anti-SPARC antibody (10 μg/mL; catalog#: AF942; R&D Systems, Minneapolis, MN, USA). Cells were then incubated in secondary antibodies (in 0.01M PBS) for 3 h at room temperature. These included donkey anti-rabbit Alexa Fluor 568 (1:200; catalog#: A10042; Invitrogen), donkey anti-goat FITC (1:100; catalog#: 705-095-147; Jackson, West Grove, PA, USA), donkey anti-chicken FITC (1:100; catalog#: 703-095-155; Jackson). Coverslips were mounted onto slides using ProLong Gold Antifade Mountant with 4′,6-diamidino-2-phenylindole (Life Technologies, Carlsbad, CA, USA). Two independent cultures (*n* = 2) and a total of 50 cells were examined per genotype. Images were acquired using a Zeiss AxioImager M2 (Zeiss, Oberkochen, Germany) microscope.

In addition, astrocyte and neuron co-cultures were processed in the same manner in order to identify co-localized VGlut2^+^ pre-synaptic and PSD95^+^ post-synaptic puncta. The following primary and secondary antibodies were used: rabbit anti-vesicular glutamate transporter 2 (VGlut2; 1:500; catalog#: 135 403; Synaptic Systems, Göttingen, Germany), mouse anti-post-synaptic density protein 95 (PSD95; 1:100; catalog#: MAB1596; Millipore), rabbit anti-GFAP (1:500; catalog#: Z0334; Dako, Burlington, ON, Canada), chicken anti-microtubule associated protein 2 (MAP2; 1:1000; catalog#: CH22103; Neuromics, Minneapolis, MN, USA), goat anti-rabbit FITC (1:100; catalog#: 111-095-144; Jackson ImmunoResearch), donkey anti-mouse Alexa Flour 594 (1:1500; catalog#: A-21203; Invitrogen), donkey anti-rabbit Alexa Flour 568 (1:200; catalog#: A10042; Invitrogen), donkey anti-chicken FITC (1:100; catalog#: 703-095-155; Jackson ImmunoResearch). Eight independent co-cultures (*n* = 4) were examined per paired condition. Co-cultures were plated on 24 well plates and wells were randomly selected for analysis (minimum 5 wells to a maximum of 17 wells were used for each *n*). Wells were discarded for analysis based on astrocyte density, only coverslips with astrocytes 70%–80% confluent were used to reduce variability among the conditions. The synapse counts were averaged across the wells to produce the value for each *n*.

### Western Blotting

Cortical and hippocampal samples containing 30 μg (homogenized whole tissue) and P14 cortical astrocyte samples containing 10 μg (isolated astrocytes) of protein were combined with 2× Laemmli Sample Buffer (Bio-Rad). Samples were heated for 5 min at 95°C, centrifuged briefly, and immediately loaded onto a gradient 4%–15% precast polyacrylamide stain-free gel (Bio-Rad) for electrophoresis. Gels intended for hevin or SPARC analysis contained age-matched WT and *Fmr1* KO samples isolated from either the whole-cortex or whole-hippocampus, and cortical astrocytes. A total of *n* = 8 samples/group were run to examine whole-cortical and -hippocampal levels of hevin and SPARC for each time-point (P7, P14 and P21) and genotype (WT and *Fmr1* KO), while a total of *n* = 4 samples/group were run to examine P14 cortical astrocyte-derived levels of hevin for each genotype (WT and *Fmr1* KO). Gels intended for FMRP analysis contained WT samples isolated from either the whole-cortex or whole-hippocampus at each time-point (P7, P14 and P21), with a total of *n* = 4–8 samples/group. Following electrophoresis, gels were activated with UV light (302 nm) for visualization of total protein (1 min) and the proteins were transferred onto polyvinyl-difluoride membranes (Bio-Rad) using the Trans-Blot Turbo Transfer System (Bio-Rad). The membranes were imaged for total loaded protein using a ChemiDoc Imaging System (Bio-Rad, Mississauga, ON, Canada), after which they were incubated for 1 h at room temperature in a 5% non-fat milk solution in Tris-buffered saline solution with Tween-20 (TBS-T). Membranes were then incubated overnight at 4°C in either anti-hevin antibody (host rabbit; 1:500; catalog#: bs-6110R; Bioss) or anti-FMRP (host rabbit; 1:1000; catalog#: 4317; Cell Signalling Technology, Danvers, MA, USA) in 5% non-fat milk/TBS-T or in anti-SPARC antibody (host goat; 0.4 μg/mL; catalog#: AF942; R&D Systems) in 2% bovine serum albumin/TBS-T). Antibodies against hevin, SPARC, and FMRP recognized bands at ~130 kDa (Figure 2B), ~37 kDa (Figure 3B), and ~80 kDa (Figure 1A) respectively. These bands representing hevin, SPARC, and FMRP were absent in negative controls incubated with only secondary antibody or an absence of primary antibody against either hevin, SPARC, or FMRP (Figures 2B, 3C). Following the incubation in primary antibody, membranes were washed in TBS-T and then incubated with horseradish peroxidase-conjugated secondary antibody against either rabbit (1:5000; catalog#: NA934-1ML; GE Healthcare Life Sciences, Mississauga, ON, Canada) or goat (1:5000; catalog#: sc-2020; Santa Cruz Biotechnology, Santa Cruz, CA, USA) in 5% non-fat milk/TBS-T for hevin detection, or in TBS-T for SPARC detection, for 1 h at room temperature. Membranes were washed again in TBS-T and developed using enhanced chemiluminescence developer solutions (Bio-Rad). Membranes were scanned using a ChemiDoc Imaging System (Bio-Rad). Densitometry measurements were conducted using Image Lab Software 5.2 (Bio-Rad). Each band corresponding to either hevin (~130 kDa), SPARC (~37 kDa), or FMRP (~80 kDa) was first normalized to total protein within the same lane, and then, if necessary, to a cross gel control. These values were then expressed as a relative percentage of the average densitometry value obtained from the age-matched WT samples.

### Synaptic Puncta Analysis

Images were obtained using a Zeiss AxioImager M2 (Zeiss, Oberkochen, Germany) microscope with Zeiss Zen Blue Imaging Software. SynapCountJ, a custom written plug-in for ImageJ (National Institutes of Health, Bethesda, MD, USA) was used to identify co-localized puncta. Thalamocortical synapse candidates were identified by the co-localization of presynaptic VGlut2^+^ and postsynaptic PSD95^+^ puncta. Cortical neurons were imaged, while thalamic neurons were avoided by the presence of intense VGlut2^+^ staining within the cell body. Low frequency background was removed from both the red and green channels of each image using the ImageJ rolling ball background subtraction algorithm. The dendrites of a neuron were traced using the ImageJ plugin NeuronJ. The coordinates of these tracings were uploaded into SynapCountJ along with the corresponding red and green channel images. The number of colocalized puncta was measured for each tracing and normalized to the tracing length.

### Statistical Analyses

Statistical analysis was conducted using GraphPad Prism Software 5.0 (GraphPad Software Inc., San Diego, CA, USA). Unpaired, two-tailed *t*-tests were used to identify significant differences in hevin and SPARC expression between WT and KO groups, using Welch’s correction when required. Significant differences in FMRP expression between the examined time-points were determined by pairwise comparisons using the nonparametric Mann-Whitney test. Paired, two-tailed *t*-tests were used to identify significant differences in thalamocortical synapse number between co-cultures containing WT and co-cultures containing KO astrocytes. All results are shown as mean ± SEM. Probability values <0.05 were considered statistically significant.

## Results

In this study, we investigated *in vivo* levels of hevin and SPARC in cortical and hippocampal brain regions of WT and *Fmr1* KO mice at ages P7, P14 and P21. Importantly, these factors are secreted by astrocytes and are important for synapse development and maturation. In FXS, dendritic spine morphology is distorted within the hippocampus and cortex (Irwin et al., 2001; Antar et al., 2006; Cruz-Martín et al., 2010), indicating abnormal development of excitatory connections with in these brain regions. We hypothesized that levels of astrocyte-derived hevin and/or SPARC may be altered in *Fmr1* KO mice and may underlie aberrant astrocyte signaling in the FXS brain. Indeed, we found that protein levels of hevin and SPARC were different in *Fmr1* KO mice compared to WT mice. While the distribution of both proteins in astrocytes maintained for 2 days *in vitro* appears consistent across the two genotypes, the overall dysregulation of these factors in *Fmr1* KO mice suggests likely contributes to the altered neurobiology in FXS.

### FMRP Expression in the Cortex and Hippocampus of WT Mice Is Developmentally Regulated

FMRP is capable of regulating the translation of many mRNAs to their corresponding proteins and can thus influence the protein milieu within the brain. Here, we assessed the developmental expression of FMRP in WT mice. Previously, hevin was identified as an mRNA target of FMRP, and thus, understanding the expression pattern of FMRP in developing WT mice may be important for understanding hevin expression patterns in *Fmr1* KO mice. FMRP in WT mice showed differential expression between time-points in both the cortex and hippocampus. FMRP expression in the cortex of WT mice was greatest at P14, and then, by P21, declined to a level less than that expressed at P7. Pairwise comparisons between time-points showed that FMRP expression at P14 was significantly greater than P21 in the cortex (P14 128.1 ± 27.50% of P7; P21 59.13 ± 12.59% of P7; *n* = 4–8/group; *P* < 0.05; Figures 1A,B). FMRP expression in the hippocampus was greatest at P7, and significantly higher than levels at P21 (P14 74.83 ± 19.77% of P7; P21 55.34 ± 13.23% of P7; *n* = 6/group; *P* < 0.05; Figures 1C,D).

### Hevin and SPARC Protein Levels Are Altered in the Cortex of *Fmr1* KO Mice

Hevin was highly expressed in primary cortical astrocytes cultured from both WT and *Fmr1* KO P1 or 2 pups, and showed a similar distribution pattern between the groups following 2 days *in vitro* (*n* = 2, 50 cells/group; Figure 2A). Western blotting revealed a difference between WT and *Fmr1* KO groups in hevin expression in cortical tissue by P14. The P14 *Fmr1* KO group showed significantly higher hevin levels than the WT group (*Fmr1* KO 144.50 ± 13.36% of WT; *n* = 8/group; *P* < 0.05; Figures 2B,D). Interestingly, there were no differences between WT and *Fmr1* KO groups at either P7 (*Fmr1* KO 81.92 ± 16.35% of WT; *n* = 8/group; Figure 2C) or P21 (*Fmr1* KO 103.80 ± 11.33% of WT; *n* = 8/group; Figure 2E) in the cortex. In order to verify that the difference observed between WT and *Fmr1* KO groups in cortical hevin levels at P14 could be attributed more specifically to differences in levels of astrocyte-derived hevin, we conducted a MACS separation to isolate astrocytes from other cell types within the cortex of both WT and *Fmr1* KO P14 mice. Consistent with our findings from whole cortical P14 tissue, hevin was expressed at higher levels in *Fmr1* KO P14 cortical astrocytes than in WT P14 cortical astrocytes (*Fmr1* KO 349.80 ± 55.78% of WT; *n* = 4/group;* P* < 0.01; Figure 2F).

In cultured cortical astrocytes derived from P1 or 2 pups, SPARC was similarly expressed between genotypes following 2 days *in vitro* (*n* = 2, 50 cells/group; Figure 3A). Representative Western blots showing SPARC (~37 kDa) from WT and *Fmr1* KO cortical samples collected at P7, P14 and P21 are shown in Figure 3B. Again, differences between groups were evident in Western blots from the different developmental time-points. In the cortex, at P7 and P14, the *Fmr1* KO group had slightly lower SPARC levels than the WT group (approximately 15% reduction at both time points; *n* = 8/group; *P* < 0.05 for both comparisons; Figures 3C,D). There was no significant difference between WT and *Fmr1* KO groups at P21 (*Fmr1* KO 94.65 ± 3.87% of WT; *n* = 8/group; Figure 3E). Thus, cortical levels of hevin and SPARC displayed differences between WT and *Fmr1* KO groups at differential developmental time-points, suggesting that altered expression of these factors during certain developmental windows contribute to aberrant synapse development in FXS.

### Hevin Protein Levels, but Not SPARC Levels, Are Altered in the Hippocampus of *Fmr1* KO Mice

**Figure 4:**
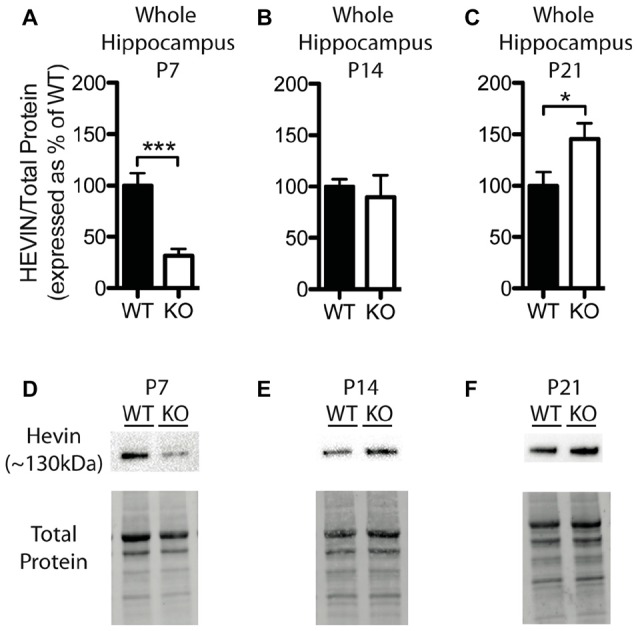
Hevin expression is altered at postnatal day (P) 7 and P21 in the hippocampus of *Fmr1* KO mice.** (A–C)** Hevin expression, determined via Western blotting, in the hippocampus of WT (black; *n* = 8) and *Fmr1* KO (white; *n* = 8) mice at P7, P14 and P21, respectively. Bands representing hevin were normalized against the total protein within the same lane on the membrane and cross gel controls, then expressed as a percent of the average level of hevin in the WT group. **(D–F)** Representative western blots show hevin (~130 kDa) in hippocampal samples (30 μg of protein per lane) from WT and *Fmr1* KO mice at P7, P14 and P21, as well as the total protein within each lane. Statistical differences were denoted with either a single asterisk, *P* < 0.05, or a triple asterisks, *P* < 0.0005.

Levels of hevin in the hippocampus differed between WT and *Fmr1* KO mice; however, these alterations were notably distinct from those in the cortex. At P7, the *Fmr1* KO group showed significantly lower hevin levels than the WT group (31.41 ± 6.86% of WT; *P* < 0.0005; *n* = 8/group; Figures 4A,D). At P14 there was no significant difference in hevin levels between *Fmr1* KO and WT groups (*Fmr1* KO 89.80 ± 21.03% of WT; *n* = 8/group; Figures 4B,E), and at P21, the *Fmr1* KO group had significantly higher hevin levels than the WT group (*Fmr1* KO 145.70 ± 15.17% of WT; *n* = 8/group; *P* < 0.05; Figures 4C,F).

**Figure 5:**
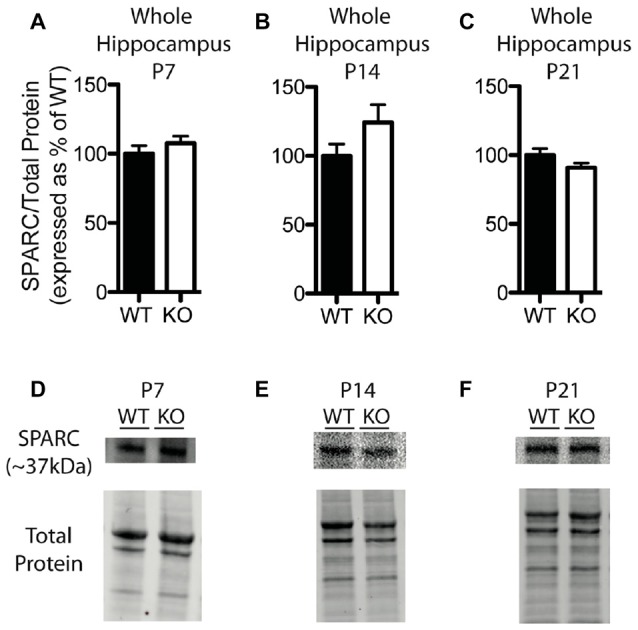
SPARC expression is not significantly altered in the hippocampus of *Fmr1* KO mice. **(A–C)** SPARC expression, determined via Western blotting, in the hippocampus of WT (black, *n* = 8) and *Fmr1* KO (white, *n* = 8) mice at postnatal day (P) 7, P14 and P21, respectively. Bands representing SPARC were normalized against the total protein within the same lane on the membrane, and were then expressed as a percent of the average level of SPARC in the WT group.** (D–F)** Representative western blots with bands at ~37 kDa corresponding to SPARC in hippocampal samples (30 μg of protein per lane) from WT and *Fmr1* KO mice at P7, P14 and P21, as well as the total protein within each lane.

In contrast to our findings with hevin expression, there were no significant differences in hippocampal SPARC levels between WT and *Fmr1* KO mice at P7 (*Fmr1* KO 107.60 ± 4.99% of WT; *n* = 8/group; Figure 5A), P14 (*Fmr1* KO 124.10 ± 12.94% of WT; *n* = 8/group; Figure 5B), or P21 (*Fmr1* KO 90.86 ± 3.26% of WT; *n* = 8/group; Figure 5C). Representative Western blots showing SPARC from WT and *Fmr1* KO P7, P14 and P21 hippocampal samples are shown in Figures 5D–F, respectively.

### The Number of VGlut2^+^/PSD95^+^ Co-Localized Puncta of WT Neurons Was Increased When Plated with *Fmr1* KO Astrocytes, Compared to Those Plated with WT Astrocytes

**Figure 6:**
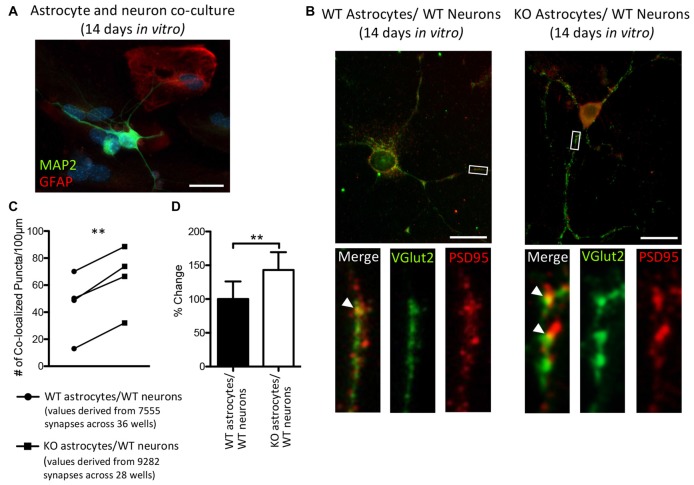
After 14 days *in vitro* the density of VGlut2^+^/ PSD95^+^ co-localized puncta is increased in co-cultures of *Fmr1* KO astrocytes and WT neurons, relative to co-cultures of WT astrocytes and WT neurons. WT cortical and thalamic neurons were isolated from P1 pups via magnetic-activated cell sorting (MACS) separation and co-cultured with either WT or *Fmr1* KO astrocytes isolated from P1 or 2 pups. Co-cultures were maintained for 14 days *in vitro*.** (A)** A co-culture with WT neurons and *Fmr1* KO astrocytes co-labeled with anti-GFAP (red) and anti-microtubule associated protein 2 (MAP2; green) to visualize astrocytes and neurons, respectively. **(B)** Co-cultures co-labeled with antibodies against vesicular glutamate transporter-2 (VGlut2) and post-synaptic density protein 95 (PSD95) to visualize pre-synaptic and post-synaptic puncta, respectively. White arrows indicate co-localized Vglut2^+^ (green) and PSD95^+^ (red) puncta. **(C)** Measures of thalamocortical synapse number (identified by the co-localized VGlut2^+^ and PSD95^+^ puncta) were obtained from cultures containing WT astrocytes (*n* = 4) and cultures containing *Fmr1* KO astrocytes (*n* = 4) and normalized to dendrite length. **(D)** The density of thalamocortical synapses in co-cultures containing *Fmr1* KO astrocytes (white) was expressed as a percentage of the density of thalamocortical synapses in co-cultures containing WT astrocytes (black). Images were obtained using a 40× objective with a Zeiss Axioimager M2. Scale bars = 25 μm. Statistical differences were denoted with a double asterisks, *P* < 0.005.

Thalamic and intracortical axonal projections that contact dendritic spines make up the majority of excitatory synapses in the cortex, and these two inputs can be distinguished by their VGlut2 or vesicular glutamate transporter-1 (VGlut1) contents, respectively (Fremeau et al., 2001; Kaneko and Fujiyama, 2002; Graziano et al., 2008). Hevin is necessary for the formation of thalamocortical excitatory synapses (Risher et al., 2014; Singh et al., 2016) and we found an increase in the cortical protein expression of hevin in P14 *Fmr1* KO mice, relative to WT mice. Therefore, we sought to determine whether a difference in the number of thalamocortical synapses would result in when WT thalamic and cortical neurons were co-cultured with either WT astrocytes or KO astrocytes (Figure 6A). Excitatory thalamocortical synaptic candidates were identified by the colocalization of VGlut2^+^ and PSD95^+^ puncta (Figure 6B). In co-cultures maintained for 14 days *in vitro* there was a 43.2% increase in the density of thalamocortical synapses when WT neurons were grown with *Fmr1* KO astrocytes (65.23 ± 11.97) relative to those grown with WT astrocytes (45.56 ± 11.88; *t*_(3)_ = 10.37, *P* < 0.005; Figures 6C,D).

## Discussion

The first few weeks of postnatal development are a time of vigorous growth, maturation, pruning, or elimination of synapses. These events must occur in a highly concerted fashion in order to establish proper synaptic connections and neuronal circuitry. Alterations in the development of synaptic structures are a hallmark of FXS (Comery et al., 1997; Irwin et al., 2000, 2001; Nimchinsky et al., 2001). Importantly, the various abnormal synapse phenotypes reported in the literature seem to be highly dependent upon the stage of development and brain region studied. Astrocytes play a significant role in the regulation of synaptic development and astrocyte dysfunction has recently been linked to neurodevelopmental disorders, such as FXS (reviewed in Sloan and Barres, 2014). Previous research from our laboratory has shown that dendrite and synapse abnormalities in cultured hippocampal neurons derived from the *Fmr1* KO mouse can be prevented by either co-culturing with WT astrocytes (Jacobs and Doering, 2010) or culturing with media conditioned by WT astrocytes (Cheng et al., 2016). Additionally, an astrocyte-specific lack of FMRP *in vivo* results in synaptic deficits within the cortex (Higashimori et al., 2016). Together, these findings suggest that aberrant astrocyte-signaling occurs in the absence of FMRP and underscore the importance of proper astrocyte-neuron interactions in the developing brain.

In this study, we examined the expression of the astrocyte-secreted factors hevin and SPARC, both of which are involved in the regulation of proper excitatory synapse development and maturation. This study is the first to investigate these factors within the context of FXS. Interestingly, we found altered levels of both hevin and SPARC in *Fmr1* KO mice compared to WT controls; however, protein expression patterns varied between the two brain regions examined. Interestingly, we found differences between WT and *Fmr1* KO groups that coincided with peak FMRP expression in the cortex (at P14; Figures 1A,B) and in the hippocampus (at P7; Figures 1C,D). These correlations may indicate time-periods during which *Fmr1* KO mice are particularly susceptible to deviations from appropriate astrocyte signaling, and thus, to the improper development of neuronal circuitry.

Normally, hevin is highly expressed in and largely restricted to astrocytes during development, and remains highly expressed in astrocytes during adulthood (Mendis et al., 1996; Cahoy et al., 2008; Eroglu, 2009). Microarray studies have shown an upregulation of *Hevin* transcripts present in the cerebellum of ASD patients (Purcell et al., 2001). Whole-genome sequencing has additionally identified possible ASD-associated mutations in* Hevin* (De Rubeis et al., 2014), which may alter the expression or function of hevin in these individuals. Here, we showed that alterations in the expression of hevin also occur in a mouse model of FXS. Perhaps this is not surprising given that hevin is a known target of FMRP (Darnell et al., 2011), but the differential expression across brain regions and developmental time points suggests that its role is not only spatially complex but also highly dependent upon temporal regulation.

In *Fmr1* KO mice, we observed a transient increase in hevin within whole cortical tissue and cortical astrocytes specifically, at age P14. Interestingly, Western blots from both the superior colliculus and whole cortical tissue homogenates have shown that hevin expression peaks at approximately P14–P25, a time-period roughly coinciding with peak synaptogenic activity (Kucukdereli et al., 2011; Risher et al., 2014). At this time intracortical and thalamocortical connections are actively being established and are not yet mature (Nakamura et al., 2005). In the cortex, excitatory synapses are primarily formed via thalamic and intracortical axonal projections that contact dendritic spines. Several lines of evidence indicate that hevin is required for the proper establishment and maintenance of thalamocortical connections. Risher et al. (2014) reported a profound reduction in thalamocortical synapses in Layer 1 of the primary visual cortex of *Hevin* KO mice at postnatal day 7, day 25 and week 12. Interestingly, this was accompanied by a transient increase of intracortical synapses at P25, a possible compensation for the reduced number of thalamocortical connections. These findings *in vivo* were supported by *in vitro* studies. When cultured cortical and thalamic neurons from *Hevin* KO mice were grown together in the presence of hevin-containing growth media there was an increase in the number of thalamocortical synapses, compared to cultures grown in media that did not contain hevin. Moreover, a subsequent study found that hevin works to establish thalamocortical connections by bridging neurexin-1 alpha and neuroligin-1B (Singh et al., 2016), two trans-synaptic molecules abundantly expressed in the brain (Schreiner et al., 2015). The adhesion between presynaptic neurexin and postsynaptic neuroligin is crucial for the establishment and maturation of synapses (Baudouin and Scheiffele, 2010). Together these studies indicate that hevin directly influences the number of thalamocortical synapses, and in doing so, may also indirectly influence the formation of intracortical synapses.

Similar to the under expression of hevin, an excess of hevin during critical developmental windows could also contribute to alterations in thalamocortical and intracortical connectivity. This possibility is consistent with findings of altered cortical function and connectivity in FXS. In the barrel cortex of 2-week-old *Fmr1* KO mice, several defects in Layer III to IV synaptic connectivity have been reported, including reduced strength, diffuse axonal arbors and altered experience-dependent plasticity (Bureau et al., 2008). The critical period for thalamocortical plasticity in the barrel cortex of mice (somatosensory layer IV), which normally occurs during the first postnatal week, is also delayed in *Fmr1* KO mice and may reflect an increase in the number of silent synapses at earlier time points (Harlow et al., 2010). Wang et al. (2014) observed an increase in the number of thalamocortical synapses in layer IV of the somatosensory cortex of 4-month-old *Fmr1* KO mice, compared to their WT counterparts. Additionally, abnormal thalamocortical connectivity has been indicated in ASD (Mizuno et al., 2006; Cheon et al., 2011; Nair et al., 2013). The increase in cortical hevin levels in P14* Fmr1* KO mice that we found, both in whole cortical tissue and in cortical astrocytes, may contribute to developmental delays in the maturation and stabilization of synapses in the cortex. Given the role of hevin in the establishment and maintainance of excitatory thalamocortical synapses (Risher et al., 2014; Singh et al., 2016), the increased density of thalamocortical synapses in cultures of *Fmr1* KO astrocytes vs. WT astrocytes found here supports the importance of hevin during this developmental window and the development of aberrant connections in the FXS cortex.

Although we also found group differences in hevin levels in the hippocampus, the pattern of hevin expression in this region was distinct from that of the cortex, suggesting an alternate mechanism by which astrocytes modulate the development of neuronal circuits in distinct brain regions. We found hevin expression in the hippocampus of P7 *Fmr1* KO mice was much lower than in WT controls, a time-point that directly coincided with maximal FMRP expression in the hippocampus (Lu et al., 2004; see also Figures 1C,D). While effects on spine and synapse phenotypes in the hippocampus of *Hevin* KO mice are unknown, pronounced deficits to excitatory synapses at P14 and P25 in the superior colliculus have been reported (Kucukdereli et al., 2011). Additionally, in Layer 1 of the primary visual cortex at P25, *Hevin* KO mice show an increase in the number of filopodia-like immature dendritic spines, concomitant with a decrease in mature spines (Risher et al., 2014). Notably, these phenotypes are similar to neurobiological abnormalities found in the hippocampus of *Fmr1* KO mice, including a reduction in the number of spines that co-localize with synaptic markers (Antar et al., 2006) and delayed synapse maturation (Braun and Segal, 2000). Reduced expression of hevin in the hippocampus, such as we observed here, may contribute to the defects in dendritic spines and synapses found in the hippocampus of *Fmr1* KO mice.

Although very low at P7, protein expression of hevin in the hippocampus of *Fmr1* KO mice increased to WT levels by P14 and exceeded them by P21. This discrepancy may be indicative of a shift in the role of hevin at these time points. Early on, hevin promotes synapse formation during postnatal development and shifts to a more regulatory role in synaptic function and plasticity during adulthood. In agreement with this, hevin has been shown to exhibit anti-adhesive properties (Gongidi et al., 2004). The presence of hevin may enhance synaptic plasticity by reducing cell adhesion and promoting spine remodeling. Additionally, hevin contains a highly conserved calcium-binding domain (Hambrock et al., 2003) and may modulate synaptic function by regulating local calcium concentrations. Indeed, more studies are needed to further elucidate the role of hevin in the brain during development and adulthood, and particularly in regard to FXS.

In addition to hevin, we examined protein levels of SPARC. SPARC is highly expressed by astrocytes in the developing brain and is capable of inhibiting the synaptogenic function of hevin (Cahoy et al., 2008; Kucukdereli et al., 2011). Due to the antagonism between SPARC and hevin, we postulated that the expression of SPARC may also differ in *Fmr1* KO mice as part of a homeostatic mechanism to compensate for alterations in hevin. However, we found only modest decreases in SPARC in the cortex of *Fmr1* KO mice at P7 and P14; and SPARC expression did not differ between genotypes at P21 in the cortex or at any time-points examined for the hippocampus. Taken together, these findings indicate that SPARC does not compensate for alterations in hevin expression. In fact, the decrease in SPARC at P14 in the cortex coincides with a robust increase in hevin, thus providing a permissive environment for the synaptogenic activity of hevin. However, more research is required to more precisely discern the mechanism by which SPARC interacts with, and regulates, the function of hevin.

## Conclusions

In this study, we found altered levels of hevin and SPARC in the *Fmr1* KO mouse that suggests aberrant astrocyte signaling in the absence of FMRP. Expression patterns of these factors differed between time-points and brain regions, implying both spatial and temporal differences in astrocyte regulatory mechanisms. These findings provide important groundwork for future studies focused on elucidating the roles of both hevin and SPARC throughout development and adulthood to help understand the mechanisms of astrocyte-derived regulation of neural circuits. Moreover, these findings emphasize the temporal and regional specificity of FXS. Identifying the functional deficits associated with aberrant levels of astrocyte-based hevin and SPARC in the FXS brain would offer important insights into novel prospects for therapeutic intervention in FXS.

## Author Contributions

JW: conception and design, collection and/or assembly of data, data analysis and interpretation, manuscript writing, final approval of manuscript. ALS: data analysis and interpretation, manuscript writing, final approval of manuscript. KR: collection and/or assembly of data, data analysis and interpretation, final approval of manuscript. LCD: conception and design, financial support, provision of study material, final approval of manuscript.

## Conflict of Interest Statement

The authors declare that the research was conducted in the absence of any commercial or financial relationships that could be construed as a potential conflict of interest.

## Abbreviations

CNO: clozapine-N-oxide
DREADDs: designer receptors exclusively activated by designer drugs
GECI: genetically encoded calcium indicators
GFAP: glial fibrillary acidic protein
GLAST: glutamate aspartate transporter
ROI: region of interest
S100β: S100 calcium-binding protein B
SERCA: sarco/endoplasmic recticulum Ca^2+^ ATPase.

## References

[B1] Allen N. J. (2013). Role of glia in developmental synapse formation. Curr. Opin. Neurobiol. 23, 1027–1033. 10.1016/j.conb.2013.06.004 23871217

[B2] Antar L. N. Li C. Zhang H. Carroll R. C. Bassell G. J. (2006). Local functions for FMRP in axon growth cone motility and activity-dependent regulation of filopodia and spine synapses. Mol. Cell. Neurosci. 32, 37–48. 10.1016/j.mcn.2006.02.001 16631377

[B3] Bakker C. E. de Diego Otero Y. Bontekoe C. Raghoe P. Luteijn T. Hoogeveen A. T.. (2000). Immunocytochemical and biochemical characterization of FMRP, FXR1P, and FXR2P in the mouse. Exp. Cell Res. 258, 162–170. 10.1006/excr.2000.4932 10912798

[B4] Bakker C. E. Verheij C. Willemsen R. van der Helm R. Oerlemans F. Vermey M. (1994). *Fmr1* knockout mice: a model to study fragile X mental retardation. Cell 78, 23–33. 10.1016/0092-8674(94)90569-x 8033209

[B5] Baudouin S. Scheiffele P. (2010). SnapShot: neuroligin-neurexin complexes. Cell 141:908. 10.1016/j.cell.2010.05.024 20510934

[B6] Beckel-Mitchener A. Greenough W. T. (2004). Correlates across the structural, functional, and molecular phenotypes of fragile X syndrome. Ment. Retard. Dev. Disabil. Res. Rev. 10, 53–59. 10.1002/mrdd.20009 14994289

[B7] Bhakar A. L. Dölen G. Bear M. F. (2012). The pathophysiology of fragile X (and what it teaches us about synapses). Annu. Rev. Neurosci. 35, 417–443. 10.1146/annurev-neuro-060909-153138 22483044 PMC4327822

[B8] Braun K. Segal M. (2000). FMRP involvement in formation of synapses among cultured hippocampal neurons. Cereb. Cortex 10, 1045–1052. 10.1093/cercor/10.10.1045 11007555

[B9] Bureau I. Shepherd G. M. G. Svoboda K. (2008). Circuit and plasticity defects in the developing somatosensory cortex of *Fmr1* knock-out mice. J. Neurosci. 28, 5178–5188. 10.1523/JNEUROSCI.1076-08.2008 18480274 PMC2696604

[B10] Cahoy J. D. Emery B. Kaushal A. Foo L. C. Zamanian J. L. Christopherson K. S.. (2008). A transcriptome database for astrocytes, neurons and oligodendrocytes: a new resource for understanding brain development and function. J. Neurosci. 28, 264–278. 10.1523/JNEUROSCI.4178-07.2008 18171944 PMC6671143

[B11] Cheng C. Lau S. K. M. Doering L. C. (2016). Astrocyte-secreted thrombospondin-modulates synapse and spine defects in the fragile X mouse model. Mol. Brain 9:74. 10.1186/s13041-016-0256-9 27485117 PMC4971702

[B12] Cheon K. A. Kim Y. S. Oh S. H. Park S. Y. Yoon H. W. Herrington J.. (2011). Involvement of the anterior thalamic radiation in boys with high functioning autism spectrum disorders: a diffusion tensor imaging study. Brain Res. 1417, 77–86. 10.1016/j.brainres.2011.08.020 21890117

[B13] Chung W. Allen N. J. Eroglu C. (2015). Astrocytes control synapse formation, function, and elimination. Cold Spring Harb. Perspect. Biol. 7:a020370. 10.1101/cshperspect.a020370 25663667 PMC4527946

[B14] Comery T. A. Harris J. B. Willems P. J. Oostra B. A. Irwin S. A. Weiler I. J.. (1997). Abnormal dendritic spines in fragile X knockout mice: maturation and pruning deficits. Proc. Natl. Acad. Sci. U S A 94, 5401–5404. 10.1073/pnas.94.10.5401 9144249 PMC24690

[B15] Cruz-Martín A. Crespo M. Portera-Cailliau C. (2010). Delayed stabilization of dendritic spines in fragile X mice. J. Neurosci. 30, 7793–7803. 10.1523/JNEUROSCI.0577-10.2010 20534828 PMC2903441

[B16] Darnell J. C. Van Driesche S. J. Zhang C. Hung K. Y. S. Mele A. Fraser C. E.. (2011). FMRP stalls ribosomal translocation on mRNAs linked to synaptic function and autism. Cell 146, 247–261. 10.1016/j.cell.2011.06.013 21784246 PMC3232425

[B17] De Rubeis S. He X. Goldberg A. P. Poultney C. S. Samocha K. Cicek E. A.. (2014). Synaptic, transcriptional, and chromatin genes disrupted in autism. Nature 515, 209–215. 10.1038/nature13772 25363760 PMC4402723

[B18] Eroglu C. (2009). The role of astrocyte-secreted matricellular proteins in central nervous system development and function. J. Cell Commun. Signal. 3, 167–176. 10.1007/s12079-009-0078-y 19904629 PMC2778595

[B19] Fremeau R. T. Jr. Troyer M. D. Pahner I. Nygaard G. O. Tran C. H. Reimer R. J.. (2001). The expression of vesicular glutamate transporters defines two classes of excitatory synapse. Neuron 31, 247–260. 10.1016/s0896-6273(01)00344-0 11502256

[B20] Gongidi V. Ring C. Moody M. Brekken R. Sage E. H. Rakic P.. (2004). SPARC-like 1 regulates the terminal phase of radial glia-guided migration in the cerebral cortex. Neuron 41, 57–69. 10.1016/s0896-6273(03)00818-3 14715135

[B21] Graziano A. Liu X.-B. Murray K. D. Jones E. G. (2008). Vesicular glutamate transporters define two sets of glutamatergic afferents to the somatosensory thalamus and two thalamocortical projections in the mouse. J. Comp. Neurol. 507, 1258–1276. 10.1002/cne.21592 18181146

[B22] Hambrock H. O. Nitsche D. P. Hansen U. Bruckner P. Paulsson M. Maurer P.. (2003). SC1/hevin: an extracellular calcium-modulated protein that binds collagen I. J. Biol. Chem. 278, 11351–11358. 10.1074/jbc.M212291200 12538579

[B23] Harlow E. G. Till S. M. Russell T. A. Wijetunge L. S. Kind P. Contractor A. (2010). Critical period plasticity is disrupted in the barrel cortex of *Fmr1* knockout mice. Neuron 65, 385–398. 10.1016/j.neuron.2010.01.024 20159451 PMC2825250

[B24] Higashimori H. Schin C. S. Chiang M. S. Morel L. Shoneye T. A. Nelson D. L.. (2016). Selective deletion of astroglial FMRP dysregulates glutamate transporter GLT1 and contributes to fragile x syndrome phenotypes *in vivo*. J. Neurosci. 36, 7079–7094. 10.1523/JNEUROSCI.1069-16.2016 27383586 PMC4938857

[B25] Irwin S. A. Galvez R. Greenough W. T. (2000). Dendritic spine structural anomalies in fragile-X mental retardation syndrome. Cereb. Cortex 10, 1038–1044. 10.1093/cercor/10.10.1038 11007554

[B26] Irwin S. A. Patel B. Idupulapati M. Harris J. B. Crisostomo R. A. Larsen B. P.. (2001). Abnormal dendritic spine characteristics in the temporal and visual cortices of patients with fragile-X syndrome: a quantitative examination. Am. J. Med. Genet. 98, 161–167. 10.1002/1096-8628(20010115)98:2<161::AID-AJMG1025>3.0.CO;2-B 11223852

[B27] Ivanov A. Esclapez M. Ferhat L. (2009). Role of drebrin A in dendritic spine plasticity and synaptic function: implications in neurological disorders. Commun. Integr. Biol. 2, 268–270. 10.4161/cib.2.3.8166 19641748 PMC2717538

[B28] Jacobs S. Doering L. C. (2009). “Primary dissociated astrocyte and neuronal co-culture,” in Protocols for Neural Cell Culture, 4th Edn. ed. Doering L. C., (New York, NY: Humana), 269–284.

[B29] Jacobs S. Doering L. C. (2010). Astrocytes prevent abnormal neuronal development in the fragile X mouse. J. Neurosci. 30, 4508–4514. 10.1523/JNEUROSCI.5027-09.2010 20335488 PMC6634485

[B30] Kaneko T. Fujiyama F. (2002). Complementary distribution of vesicular glutamate transporters in the central nervous system. Neurosci. Res. 42, 243–250. 10.1016/s0168-0102(02)00009-3 11985876

[B31] Kronk R. Bishop E. E. Raspa M. Bickel J. O. Mandel D. A. Bailey D. B. Jr. (2010). Prevalence, nature, and correlates of sleep problems among children with fragile X syndrome based on a large scale parent survey. Sleep 33, 679–687. 10.1093/sleep/33.5.679 20469810 PMC2864883

[B32] Kucukdereli H. Allen N. J. Lee A. T. Feng A. Ozlu M. I. Conatser L. M.. (2011). Control of excitatory CNS synaptogenesis by astrocyte-secreted proteins Hevin and SPARC. Proc. Natl. Acad. Sci. U S A 108, E440–E449. 10.1073/pnas.1104977108 21788491 PMC3156217

[B33] Lu R. Wang H. Liang Z. Ku L. O’Donnell W. T. Li W.. (2004). The fragile X protein controls microtubule-associated protein 1B translation and microtubule stability in brain neuron development. Proc. Natl. Acad. Sci. U S A 101, 15201–15206. 10.1073/pnas.0404995101 15475576 PMC524058

[B34] Lubs H. A. Stevenson R. E. Schwartz C. E. (2012). Fragile X and X-linked intellectual disability: four decades of discovery. Am. J. Hum. Genet. 90, 579–590. 10.1016/j.ajhg.2012.02.018 22482801 PMC3322227

[B35] Marco E. J. Hinkley L. B. Hill S. S. Nagarajan S. S. (2011). Sensory processing in autism: a review of neurophysiologic findings. Pediatr. Res. 69, 48R–54R. 10.1203/PDR.0b013e3182130c54 21289533 PMC3086654

[B36] Mendis D. B. Shahin S. Gurd J. W. Brown I. R. (1996). SC1, a SPARC-related glycoprotein, exhibits features of an ECM component in the developing and adult brain. Brain Res. 713, 53–63. 10.1016/0006-8993(95)01472-1 8724975

[B37] Mizuno A. Villalobos M. E. Davies M. M. Dahl B. C. Müller R. A. (2006). Partially enhanced thalamocortical functional connectivity in autism. Brain Res. 1104, 160–174. 10.1016/j.brainres.2006.05.064 16828063

[B38] Nair A. Treiber J. M. Shukla D. K. Shih P. Müller R. A. (2013). Impaired thalamocortical connectivity in autism spectrum disorder: a study of functional and anatomical connectivity. Brain 136, 1942–1955. 10.1093/brain/awt079 23739917 PMC3673456

[B39] Nakamura K. Hioki H. Fujiyama F. Kaneko T. (2005). Postnatal changes of vesicular glutamate transporter (VGluT)1 and VGluT2 immunoreactivities and their colocalization in the mouse forebrain. J. Comp. Neurol. 492, 263–288. 10.1002/cne.20705 16217795

[B40] Nimchinsky E. A. Oberlander A. M. Svoboda K. (2001). Abnormal development of dendritic spines in *FMR1* knock-out mice. J. Neurosci. 21, 5139–5146. 11438589 10.1523/JNEUROSCI.21-14-05139.2001 PMC6762842

[B41] Pacey L. K. K. Doering L. C. (2007). Developmental expression of FMRP in the astrocyte lineage: implications for fragile X syndrome. Glia 1609, 1601–1609. 10.1002/glia.20573 17823967

[B42] Pfeiffer B. E. Huber K. M. (2009). The state of synapses in fragile X syndrome. Neuroscientist 15, 549–567. 10.1177/1073858409333075 19325170 PMC2762019

[B43] Purcell A. E. Jeon O. H. Zimmerman A. W. Blue M. E. Pevsner J. (2001). Postmortem brain abnormalities of the glutamate neurotransmitter system in autism. Neurology 57, 1618–1628. 10.1212/WNL.57.9.1618 11706102

[B44] Risher W. C. Patel S. Kim I. H. Uezu A. Bhagat S. Wilton D. K.. (2014). Astrocytes refine cortical connectivity at dendritic spines. Elife 3:e04047. 10.7554/eLife.04047 25517933 PMC4286724

[B45] Schreiner D. Simicevic J. Ahrné E. Schmidt A. Scheiffele P. (2015). Quantitative isoform-profiling of highly diversified recognition molecules. ELife 4:e07794. 10.7554/eLife.07794 25985086 PMC4489214

[B46] Sidorov M. S. Auerbach B. D. Bear M. F. (2013). Fragile X mental retardation protein and synaptic plasticity. Mol. Brain 6:15. 10.1186/1756-6606-6-15 23566911 PMC3636002

[B47] Singh S. K. Stogsdill J. A. Pulimood N. S. Dingsdale H. Kim Y. H. Pilaz L. J.. (2016). Astrocytes assemble thalamocortical synapses by bridging NRX1α and NL1 via hevin. Cell 164, 183–196. 10.1016/j.cell.2015.11.034 26771491 PMC4715262

[B48] Sloan S. A. Barres B. A. (2014). Mechanisms of astrocyte development and their contributions to neurodevelopmental disorders. Curr. Opin. Neurobiol. 27, 75–81. 10.1016/j.conb.2014.03.005 24694749 PMC4433289

[B51] Wang T. Bray S. M. Warren S. T. (2012). New perspectives on the biology of fragile X syndrome. Curr. Opin. Genet. Dev. 22, 256–263. 10.1016/j.gde.2012.02.002 22382129 PMC3653273

[B50] Wang H. Ku L. Osterhout D. J. Li W. Ahmadian A. Liang Z. (2004). Developmentally-programmed FMRP expression in oligodendrocytes: a potential role of FMRP in regulating translation in oligodendroglia progenitors. Hum. Mol. Genet. 13, 79–89. 10.1093/hmg/ddh009 14613971

[B49] Wang G. X. Smith S. J. Mourrain P. (2014). *Fmr1* KO and fenobam treatment differentially impact distinct synapse populations of mouse neocortex. Neuron 84, 1273–1286. 10.1016/j.neuron.2014.11.016 25521380 PMC4479348

